# Sustainable Machining of Mg-9Al-1.4Zn Foam Used for Temporary Biomedical Implant Using Cryogenic Cooling

**DOI:** 10.3390/ma15196678

**Published:** 2022-09-26

**Authors:** Abdalla Mohammed, Sathish Kannan, Salman Pervaiz, Shafahat Ali, Kevin K. Thomas, Ramanujam Karthikeyan

**Affiliations:** 1 Department of Mechanical Engineering, American University of Sharjah, Sharjah P.O. Box 26666, United Arab Emirates; 2 Department of Mechanical Engineering, Rochester Institute of Technology, Dubai P.O. Box 341055, United Arab Emirates; 3 Department of Mechanical Engineering, BITS Pilani, Dubai Campus, Dubai P.O. Box 345055, United Arab Emirates

**Keywords:** hole drilling, surface integrity, machining forces, Mg-9Al-1.4Zn foam

## Abstract

In this study, the drilling performance of biodegradable grade Mg-9Al-1.4Zn alloy reinforced with hollow thin-walled Al_2_O_3_ microspheres is inspected under different coolant environments such as dry, Almag^®^ mineral oil, and liquid nitrogen. Drilling experiments were carried out using titanium aluminum nitride PVD coated and uncoated K10 tools on varying volume fractions of magnesium syntactic foams (5%, 10%, and 15%) reinforced with hollow Al_2_O_3_ microspheres. Test results showed a 30–60% higher thrust force generated with liquid nitrogen drilling in comparison to dry and oil-based drilling while cutting higher volume fraction foams. Higher microsphere volume fractions of syntactic foam recorded higher machining forces, which is roughly a 200% increase as the volume fraction raised to 15%. The performance of TiAlN PVD tool coating is reflected through a reduction in thrust forces by 20% during cryogenic drilling. Scanning electron microscope (SEM) investigation of cryogenic-machined bore surfaces showed minimal drilling-induced surface defects compared to dry and Almag^®^ mineral oil conditions. A three-dimensional, thermo-mechanical finite element-based model for drilling Mg-9Al-1.4Zn syntactic foam using AdvantEdge^TM^ is developed for different sustainable lubrication conditions. Surface finish (Ra) showed a 45–55% improvement during cryogenic drilling of 15% syntactic foams with minimized subsurface damages compared to dry and wet cutting conditions. The higher the volume fraction, the higher the surface roughness (Ra) and thrust force under cryogenic machining.

## 1. Introduction

Mg-9Al-1.4Zn bioresorbable syntactic foams are a special class of material system where the presence of hollow microspheres enhances their corrosion resistance and make them a durable material that is ideal for biomedical temporary implant applications. Mg-9Al-1.4Zn-based hollow alumina syntactic foams due to their superior corrosion resistance and mechanical strength find potential uses in biomedical applications. This unique biodegradable composition is near to the mechanical properties of human bone and is found to be an attractive material to be used as temporary orthopedic implants [1]. Due to the rapid degradation of magnesium and its alloys in biological fluid, it loses its mechanical integrity and fails to perform before the complete healing of bone fracture (in orthopedics application) or removal of plaque in arteries (in the case of vascular implants). Using suitable alloying elements, mechanical strength and corrosion resistance of Mg-alloys can be enhanced, but cytotoxicity and long-term inflammatory consequences of these elements are a major concern. Further modifying the surface characteristics of Mg-alloy through various surface coating, machining, mechanical working, etc., corrosion behavior can be manipulated [2]. Surface texturing using an Nd:YAG laser has been employed on pure Mg aimed with less success, primarily aimed at creating localized texturing or remelting areas to enhance its biocorrosion performance [3].

During the past years, an increase in the number of people choosing to undergo orthopedic implantation surgical operations has been noticed. These materials possess properties such as elastic modulus, density, and mechanical properties close to human bones that promote tissue generation and aid in the healing process. These materials are starting to play a critical role in the design and manufacture of orthopedic and craniofacial fixations [1]. This also removes the need for a second operation and helps in the recovery of damaged tissues. However, magnesium and its alloys have high biodegradation/corrosion rates, especially at the early stage of implantation in the human body. The cell surfaces surrounding the implant material are influenced by a higher pH of the fluid where higher concentrations of hydroxyl anions are noticed [4]. The main degradation products of magnesium are required for several biological enzymic activities and in the formation of bone cells through improved osteoinduction and antibacterial properties [5,6]. However, in order to improve the corrosion resistance of these materials, an appropriate percentage of Al is added to the Mg-Zn alloy, which shows superior performance [4].

Magnesium-based syntactic foams are classified as hard to machine due to the presence of hollow alumina microspheres [7,8]. A key challenge faced during the drilling of Mg-9Al-1.4Zn syntactic foams is due to edge delamination and surface damage [8,9]. In addition to this, other key defects include built-up edge, surface residual stress, and porosity on machined surfaces [7]. Surface modifications of biomaterials play a vital role in matching the complexities of the biological system and improving the performance of bioimplants. Suitably customized surface modifications can substantially enhance the relations between materials toward biocompatibility, bondability, and host cell associations. In addition, material surface engineering accelerates the progression and configuration of next-generation biomaterials and restorative gadgets [10]. Past studies have shown a strong correlation between machined surface integrity and its influence on corrosion performance during machining magnesium alloys [11]. The rapid degradation of surface properties due to machining-induced defects could restrict their clinical applications. Machining with large radii cutting tools under a liquid nitrogen cooling medium shows a positive impact on the subsurface integrity of magnesium alloys [12,13]. Pereira et al. [14] showed that liquid nitrogen coolant reduces tool wear by 40% before investigating liquid carbon dioxide. Pereira et al. also found that liquid nitrogen coolant decreases the adhesive and diffusive wear on the cutting tool, and prevents plastic deformation of the tool [15]. Reducing the contact area between the cutting tool rake surface and chip promotes the machining performance of the work material and increases the tool’s life. Magnesium alloys are ductile-lightweight materials that form continuous chips during machining. In the case of cryogenic machining, textured tools substantially minimize the friction by the coupled effect of micro-pool lubrication and the formation of thin-film lubrication between the tool–chip/tool–work interfaces. Parallel-textured tools aided with cryogenic cooling exhibit superior performance during machining among the different types of tools employed in the cryogenic machining of biodegradable ZK60 Mg alloy using micro-textured tools [16]. In a cutting test on AZ31 magnesium alloy using a dipped cryogenic drilling method, Ugur et al. [17] reported a rise in thrust force. However, cryogenic machining was shown to produce smaller chips with lower material adhesion.

During the machining of AZ91D MMC, graphite reinforcement particles provide a useful medium of lubrication that reduces the surface roughness [18]. The drilling feed rate is found to be a dominant factor in controlling machined surface roughness. The higher the feed rate, the more severe the surface anomalies with grooves and cracks caused by the strengthening of the matrix phase [19]. During the micro-drilling of magnesium-silica nanocomposites, the chip morphology changed from short spiral type to powder type [20]. Surface quality reduced with higher values of volume fraction, and feed rate with larger burrs formed [20]. In a study on machining magnesium-based metal composites, the chip shape is found to be dependent on the cutting velocity [21].

Sustainable machining processes such as cutting in dry conditions, MQL, and LN_2_ lubrication are important research areas for magnesium composite foams. During the cryogenic drilling of AZ31B magnesium alloy, an increase in machined surface hardness is noted [22,23]. On the other hand, the minimum quantity lubrication (MQL) is shown to produce a stable thrust force during the cutting of the AM60 Mg alloy. The MQL method generates acceptable surface quality with the formation of discontinuous chips. A method to predict the surface roughness during MQL drilling of Mg material has been developed [24]. A point angle of 118° is reported to produce a better surface finish at high cutting speeds under MQL conditions.

Dry machining of magnesium alloys leads to poor surface finish, and material adhesion to the cutting tool results in surface smearing and reduction in tool life [25]. This is particularly important during drilling operations, which could lead to the poor evacuation of chips from the drilled hole. On the other hand, dry machining can also be beneficial from an environmental perspective, provided the process parameters are optimized. There is a need to control the cutting velocity to prevent the sticking of soft magnesium on the tool [26,27]. During the drilling of magnesium alloy in dry condition, Wang et al. [28] developed wear mechanisms maps in which they identified five different zones. Bhowmick et al. [29] reported accelerated adhesive tool wear during dry machining of an as-cast magnesium alloy. However, the situation was improved while using diamond-like carbon high-speed tools under MQL conditions. Drilling tests conducted on Mg-Al alloy using water-based MQL and a fatty acid-based MQL showed a reduction in thrust forces, cutting temperature, and better surface finish [29].

Several studies have been carried out to understand the effect of tool properties and cutting factors on the machinability of porous tungsten [30,31]. In their study, Schoop et al. [31] obtained a suitable porous surface by controlling tungsten brittle microfracture. Heidari et al. [32] showed good quality surface roughness despite open porosity. Pusavec [33] used a multi-objective optimization mode via a generic algorithm for the cryogenic machining of cutting porous titanium.

The development of sustainable cooling methods for machining magnesium-based composites is critical from the perspective of mass production of biomedical orthopedic implants. This paper investigates the performance of sustainable cooling methods during the hole drilling of Mg-9Al-1.4Zn-based alumina syntactic foams. The different cooling methods have been compared for their ability to produce a hole in terms of surface quality and integrity, burr formation, and drilling forces generated.

For a widespread application of these novel material systems and transforming them from their near-net shapes into a useful final product will require the development of sustainable machining methods. The biomedical products manufactured will require extensive conventional machining operations, such as drilling, tapping, reaming, and milling for producing features such as bolt holes, threads, screws, grooves, slots, etc. The temporary biomedical implants comprise several threaded holes, slots, pockets, contour milling, profiles, etc., which require the highest quality of surface finish and integrity with minimal machining-induced defects. Unconventional processes such as LASER or electric discharge machining (EDM) could cause heat-affect zones and unnecessary white layers on the machined implant surfaces. These processes may not be appropriate for finish machining these surface-sensitive biomedical products, which may affect their functional performance as bone implants inside the human body (e.g., accelerated corrosion). This could even result in implant loosening or even catastrophic damage requiring a second operation causing severe discomfort to the patient.

The literature available till today has concentrated mostly on statistical analysis and experimental investigation of various parameters during foam drilling operations. In this paper, an attempt has been made to develop sustainable lubrication methods to minimize the machining-induced defects on the Mg-9Al-1.4Zn-based alumina syntactic foams. These defects could be related to the corrosion rate and degradation of the material. These materials are being studied for use on temporary orthopedic implants where superior surface quality and integrity are sought. To understand the varied behavior exhibited by samples under different sustainable lubrication methods, a thorough characterization of the machined surface integrity, including microstructural observations, macro and microcracking assessments, and machined texture analysis has been carried out. The study is complemented through the development of a three-dimensional, thermomechanical finite element simulation for modeling the machining behavior of Mg-9Al-1.4Zn-based alumina syntactic foams.

This study used various sustainability techniques to examine the behavior of different cutting conditions. Since magnesium alloys have low flash points, cryogenic conditions can be used to improve machining performance over dry cutting [17,19]. Various cutting parameters are varied to observe their effect on responses, such as corrosion rate, cryogenic over wet oil and dry machining. Different machining processes have been used to machine magnesium alloy for biomedical implants, including drilling [13,17,19]. It has been used in diverse biomedical implants, including bone plates and other struts in different parts of the human body [5]. A magnesium foam has been studied in this work because of its enhanced biocompatibility and biodegradability [5,11].

## 2. Material and Methods 

Mg-9Al-1.4Zn syntactic foams reinforced with varying volume fractions of hollow thin-walled alumina microspheres were manufactured via the stir-squeeze casting method. The melt was stirred at 500 rpm for 10 min while the alumina microspheres were added. The melt temperature was set at 750 °C with the mold preheated to 300 °C under inert pure argon gas at 3 liters per minute. The electromagnetic vibrator was used at 320 Hz to distribute the hollow microspheres, which were preheated to 200 °C into the melt. The squeezing pressure was 117 MPa, which produced a billet of 50 mm diameter and 200 mm long. Cylindrical billets were manufactured by SwamEquip Ltd., Chennai, India. The chemical composition of hollow alumina was obtained from Pacific Rundum Co., Ltd, Tokyo, Japan. The composition for the hollow alumina microspheres and the matrix is summarized in Table 1.

### 2.1. Twist Drills

**Figure 1:**
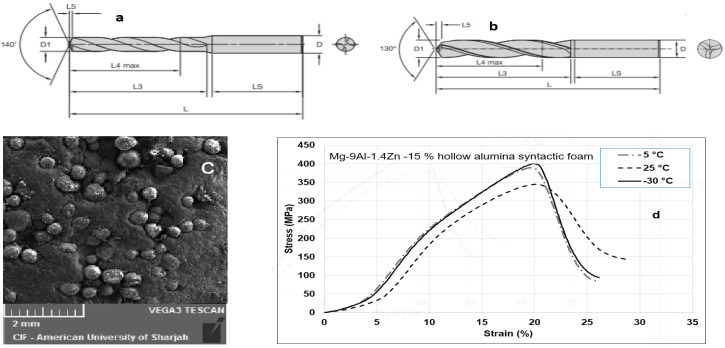
Kennametal™ twist drills: (**a**) TiAlN PVD coated. (**b**) Uncoated carbide. (**c**) Representative microstructure of Mg-9Al-1.4Zn -15% hollow alumina syntactic foam. (**d**) Compression stress–strain curve for Mg-9Al-1.4Zn -15% hollow alumina syntactic foam @ strain rate: 1 s^−1^.

Two grades of carbide twist drills were used in this study. A multilayer titanium aluminum nitride (TiAlN)-coated carbide drill, and uncoated K10 carbide drills were procured from Kennametal^TM^. Both the drill bits are standard off-the-shelf tools used for machining magnesium alloys. The specifications for the tool are shown in Figure 1a,b. The twist drill coated with TiAlN has superior wear and heat resistance using the physical vapor deposition method. Both the twist drills were Ø5 mm in diameter. Properties of the twist drills used in this study are shown in Table 2.

### 2.2. Drilling and Lubrication Conditions

**Figure 2:**
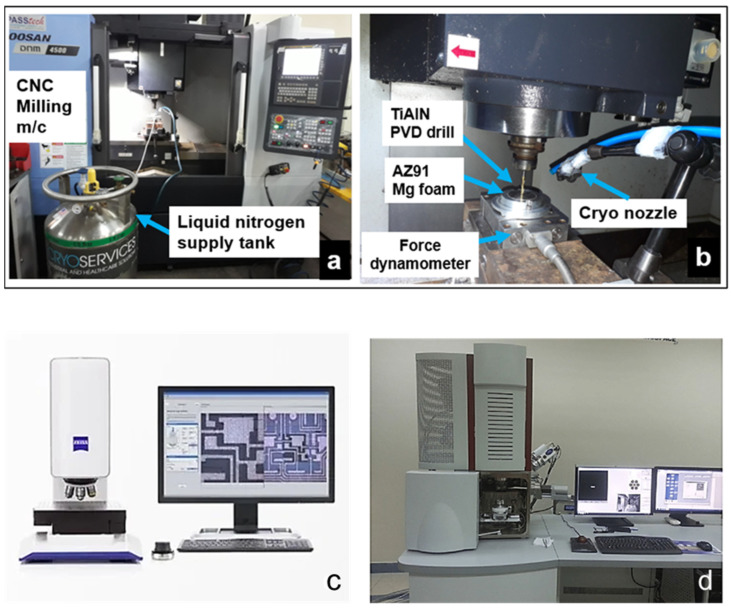
(**a**) Doosan machine tool. (**b**) Drilling process set up. (**c**) ZEISSS smart proof 5 microscope. (**d**) TESCAN SEM setup.

Cutting assessments were performed on a Doosan DNM-4500 3-axis milling machine. Three sustainable cooling methods were tested. Machining-induced surface defects were characterized while cutting under dry, liquid nitrogen cryogenic, and mineral oil-based coolant plunge drilling conditions. Machining conditions experimented on are shown in Table 3. Five-millimeter-deep holes were drilled on magnesium syntactic foam samples reinforced with 5%, 10%, and 15% volume fractions of hollow Al_2_O_3_ microspheres. Average values of machining force, surface roughness, and burr heights were recorded based on the three holes drilled. Chip shapes were investigated under a Tescan electron microscope. The functional performance of biodegradable Mg-9Al-1.4Zn-based alumina syntactic foams considered for advanced engineering applications requires exceptional surface quality and integrity requirements. The condition of the machined surface also controls the corrosion performance of the manufactured products [19]. This demands the need to employ favorable lubrication conditions to meet this requirement. As part of sustainable manufacturing, dry machining helps to reduce waste and product costs. The benefits of dry machining are explored in this study. Compressed air was used during dry cutting to clear the machined loose chips adhered to the hole surface, thereby preventing the ignition of magnesium chips. Mineral oil-based cutting fluid Almag^®^ was selected for the test due to its low viscosity and is the preferred choice for the machining of magnesium alloys. The absence of water in this product prevents the formation of heat and explosive hydrogen while cutting magnesium foams. It has been shown that cryogenic machining is more suitable for producing favorable surface residual stresses during machining AZ31-O magnesium alloys [19]. In this study, liquid nitrogen-based cryogenic cooling (pressure-3 bar) was applied through a delivery hose (Figure 2a,b).

### 2.3. Machining Force, Surface Roughness, Burr Formation, and Chip Morphology

A KISTLER™ 9129AA three-channel dynamometer (Kistler Instrument Corp., Novi, MI, USA) was used along with a multichannel charge amplifier type 5080 to measure the drilling forces (uncertainty ± 20 N). In this study, only the thrust forces were recorded as the capability to measure torque was not available. A ZEISS Smartproof^TM^ (Carl Zeiss Microscopy, White Plains, NY, USA) confocal surface analyzer was employed to investigate the bore surface roughness (Ra) on different locations of the hole as shown in Figure 2c. Average values were noted for each test condition. The machining-induced surface defects were characterized using a TESCAN SEM (TESCAN ORSAY HOLDING, Kohoutovice, Czech Republic) for different cooling conditions. During the machining of magnesium-based foams, burrs are formed due to material side flow [7,8]. In this work, the influence of the cooling method and volume fraction of hollow Al_2_O_3_ in the foam on the burr shape formed was investigated. The burr height was measured using a ZEISS Smartproof^TM^ confocal microscope, and their shapes were studied using an SEM. Chip morphology was studied using the chips collected from each test, which were cleaned in ethanol before SEM analysis.

The universality of the applied methodology will be generalized and can be applied to all kinds of metal syntactic foams (Metal matrix and Metal syntactic foam types). Several parameters have been selected from different perspectives. However, the maximum percentage used in this experiment is 15% to prevent the material from behaving more brittle, which is not recommended for biomedical applications. In this experimentation, a wide range of feed rates have been studied to see their impact on the machining performance. Different coolant conditions and cutting tools are used to reduce the cutting temperature in the cutting zone due to the magnesium alloy’s low flash point and to improve the machining characteristics.

### 2.4. AdvantEdge^TM^ Model

A finite element (FE) machining simulation program was developed using AdvantEdge^TM^ (7.7, Third Wave Systems, Eden Prairie, MN, USA). The cutting conditions and tool geometry used in the simulations were the same as those used in the experiments (Table 2 and Table 3). In this software, Johnson–Cook constitutive material model is used to describe the flow stress of the Mg-9Al-1.4Zn-magnesium matrix [8]. To simulate the drilling forces, the workpiece was assumed as a homogenous isotropic material using 3 node triangle elements. The material model was assumed as a homogenous model. This assumption is the main limitation of the AdvantEdge^TM^ machining software, whereby multi-material alloys/composites cannot be modeled. Hence, the authors collected the physical and mechanical properties of the foam, and modeled them as a single homogenous foam material. Although this machining software does not allow for any micro-failure mechanisms such as debonding and bubble fracture to be analyzed, the overall magnitude of cutting forces and stress distributions for the modeled material was satisfactorily predicted.

A yield surface material model is used in this study to model magnesium alloy using Johnson and Cook (JC) constants to model material stress. Equation (1) below gives the constitutive model.

(1) $$\sigma =\left[R+S{\left(\varepsilon \right)}^{x}\right]\left[1+W\ln\left(\frac{\varepsilon }{\varepsilon_{0}}\right)\right]\left[1-{\left(\frac{T-T_{ref}}{T_{m}-T_{ref}}\right)}^{y}\right]$$

**Figure 3:**
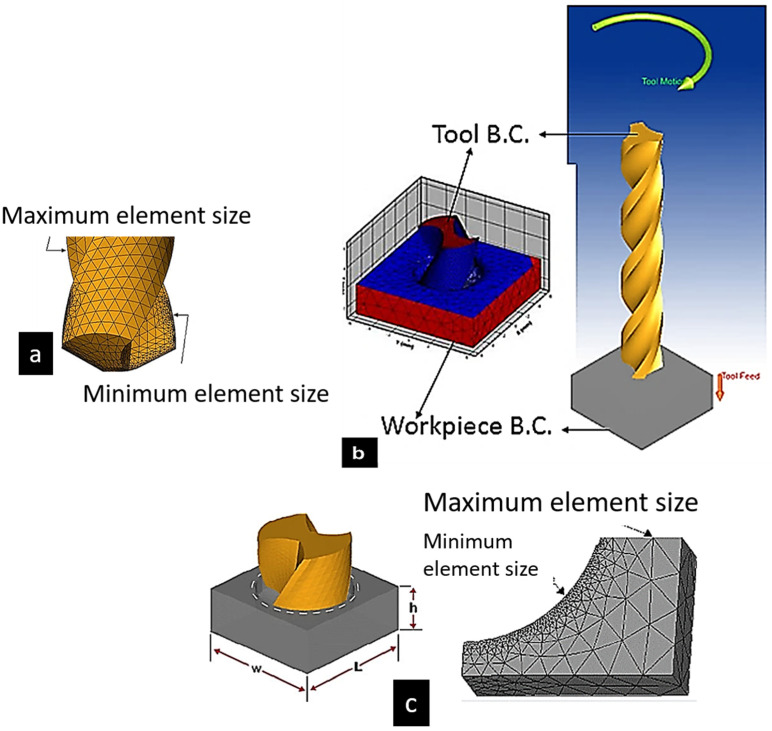
(**a**) FE tool in mesh condition. (**b**) Workpiece and tool boundary conditions. (**c**) Workpiece under meshing condition.

Material constants include R, S, W, x, and y. The constant values, where S (605 MPa) is the strain hardening constant, x (0.52) is the strain hardening coefficient, R (140 MPa) is the yield stress, W (0.03 s^−1^) is the strengthening coefficient of strain rate, y (0.32) is the thermal softening coefficient, $T_{ref}$ (8 °C) is the room temperature, $T_{m}$ (533 °C) is the melting temperature of the material experiencing plasticity, and $\varepsilon_{0}$ (1 s^−1^) is the reference strain rate [8]. There are different parameters used in the modeling of this experimentation where design parameters of the tool such as the point angle as 140 degrees, chisel edge angle as 120 degrees, and clearance angle as 9 degrees. In addition, the characteristics of the drill bit are as follows: helix angle of 30 degrees, flute radius and length of 1.45 mm and 20 mm, and edge radius of 0.04 mm. Furthermore, the cutting tool’s minimum and maximum element sizes were 0.03 mm and 0.3 mm, with a mesh grading of 0.4. Different criteria were set in the software for the workpiece. The minimum and maximum element sizes were 0.022 mm and 1.5 mm, with a mesh grading and resolution factor of 0.2 and 3, respectively. The workpiece thickness was 5 mm, and both thickness and length were 12 mm. Figure 3 shows the model of the workpiece used in the software.

In AdvantEdge, a chip separation criterion employs a critical value to estimate when chip separation will begin depending on physical conditions. The correlation between damage and effective plastic displacement ($DE^{pl}$) is believed to be linear. The plastic displacement at the point of failure ($DE_{f}^{pl}$) can be entered into the equation below such that it grows in proportion to the displacement (*d*) at the point of failure. When the damage variable is equal to 1, the chip separation requirements are enabled by material failure [34].

(2) $$d=\frac{DE^{pl}}{DE_{f}^{pl}}$$

A coefficient of friction is determined by the relationship between sliding friction force and normal-to-friction force in AdvantEdge^TM^. The coefficient is uniform in all secondary shear zones between the chip and the cutting tool. AdvantEdge^TM^ provides friction conditions based on the Coulomb friction model [34], whose equation is:

(3) $$F_{FR}=\mu \times N$$

This equation consists of $F_{FR}$ as sliding friction force, N as normal-to-friction force, and the normal load acting perpendicularly to the direction of sliding friction force and μ as the friction coefficient.

## 3. Results and Discussion

### 3.1. Thrust Force

**Figure 4:**
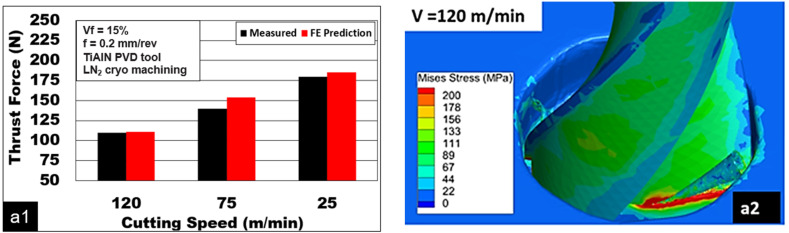

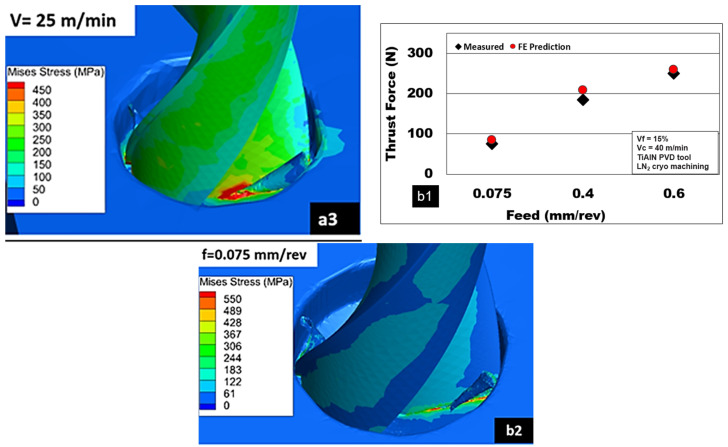
Effect of process parameters during machining Mg-9Al-1.4Zn-15% hollow Al_2_O_3_ foam: (**a1**) effect of cutting speed, comparison between experiment and FE prediction; (**a2**,**a3**) von Mises stress distribution for V_C_ = 125 m/min and V_C_ = 25 m/min; (**b1**) force comparison between experiment and FE prediction for feed conditions; (**b2**) von Mises stress distribution for feed = 0.075 mm/rev (coated drill, cryogenic cooling).

Figure 4(a1) shows the variation in thrust forces generated during the drilling of Mg-9Al-1.4Zn syntactic foams reinforced with 15% hollow Al_2_O_3_. The cutting test was carried out using TiAlN PVD-coated twist drills under cryogenic cooling conditions. Thrust forces increased by 64% from 110 to 180 N with a decrease in drilling speed from 100 to 25 m/min. Through mechanical testing, Mg-9Al-1.4Zn foam is shown to undergo brittle fracture [8]. At higher cutting speeds, the higher rate of shear loading acts on the material. This induces the thermal softening of the magnesium matrix and ceramic hollow microspheres to be fractured. A preferential propagation of brittle cracks along the shear zone could result. A reduction in shear plane length, chip-tool contact area, and friction force are expected at higher cutting speeds. This could be the possible reason for the reduction in thrust force. Higher magnitudes of von Mises stress distribution are noted for lower cutting speeds (Figure 4(a2,a3)). Figure 4(b1) shows the thrust force generated while cutting Mg-9Al-1.4Zn-based Al_2_0_3_ syntactic foams at a range of feed values and at 40 m/min cutting speed. Machining using a TiAlN PVD-coated drill under liquid nitrogen cooling conditions showed an increase in thrust force values by 175 N as the drilling feed raised to 0.6 mm/rev. This is an almost 233% increase in the magnitude of thrust forces, which was recorded as 75 N and 250 N at lower and higher feed values. Figure 4(b1) also shows the force comparison between the experiment and FE prediction for different feed conditions. Figure 4(b2) shows the von Mises stress distribution for feed = 0.075 mm/rev (coated drill, cryogenic cooling). An increase in chip-tool contact area causes a higher magnitude of thrust force. The role played by hollow microspheres in controlling the work-hardening behavior of the magnesium matrix is primarily shown as the reason for the higher force values [35].

**Figure 5:**
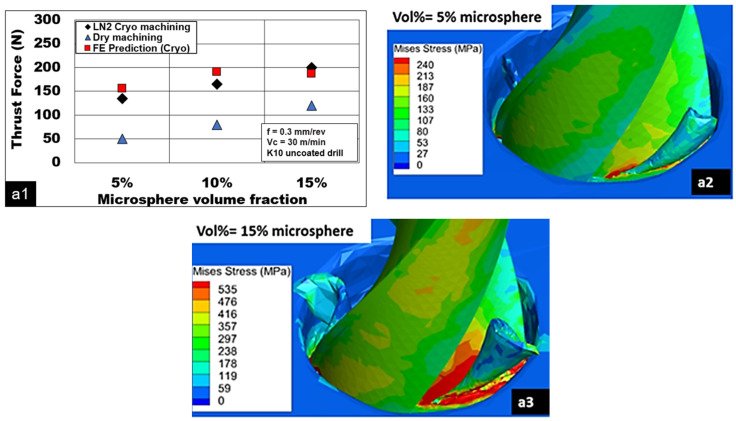
(**a1**) Effect of alumina volume fraction on thrust force during machining Mg-9Al-1.4Zn reinforced with varying vol% of hollow alumina microspheres, and (**a2**,**a3**): von Mises stress distribution for Mg 5% and 15% hollow alumina foams. (V = 30 m/min, f = 0.3 mm/rev, K10-uncoated drill, cryogenic cooling).

The role of the volume fraction of hollow Al_2_O_3_ on thrust forces generated during cutting Mg-9Al-1.4Zn-based Al_2_O_3_ syntactic foam is shown in Figure 5(a1). An increase in the volume fraction of Al_2_O_3_ increases their number in the Mg-9Al-1.4Zn matrix. Along with second phase precipitates of Al and Zn, the role played by the ceramic alumina reinforcements in enhancing the work-hardening behavior of the magnesium matrix is significant [7]. The presence of these reinforcements affects the extent of the plastic deformation of these foams through a characteristic load transfer that enables them to improve their peak strength. It is reported that the higher the number of hollow Al_2_O_3_ in the magnesium, the lower the plasticity of foam will be [7]. A reduced ductility and strain to fracture are reported with a higher volume fraction of hollow microspheres, indicating an increase in its brittleness [7,8]. While cutting Mg-9Al-1.4Zn-based Al_2_O_3_ syntactic foams under the cryogenic cooling method, an increase in thrust force by 50% while cutting with an increasing volume fraction (15%) is observed. Figure 5(a2,a3) show the distribution of Von Mises stress. At a lower volume fraction of hollow Al_2_O_3_, drilling thrust forces are in the range of 135 N, which increased to around 200 N while cutting magnesium foam with 15% vol of alumina. This increase in thrust force is primarily attributed to the increase in peak compressive strength of the magnesium foam with an increasing volume fraction of hollow Al_2_O_3_ microspheres. However, during dry machining, the increase in thrust force recorded was almost 200% when the volume fraction of hollow alumina was increased from 5% to 15%. In general, it is observed that thrust forces generated during dry cutting were lower than the cryogenic machining for all volume fraction magnesium foams considered in this study. For 5% volume fraction Mg-9Al-1.4Zn foams, the thrust force generated during cryogenic machining was 170% higher than dry machining. Peak cutting temperature reduces, resulting in higher thrust forces. However, as the volume fraction of hollow alumina increased to 15%, the force margin reduced to 35%. This phenomenon indicates the increasing brittleness with increasing hollow Al_2_O_3_ microspheres and their influence on the hardening behavior of the Mg-9Al-1.4Zn matrix. Although the thrust forces were also measured under dry machining, it was measured with great difficulty owing to chances for chip ignition [36]. So, FE prediction thrust forces were preferred for cryogenic machining instead of dry machining.

**Figure 6:**
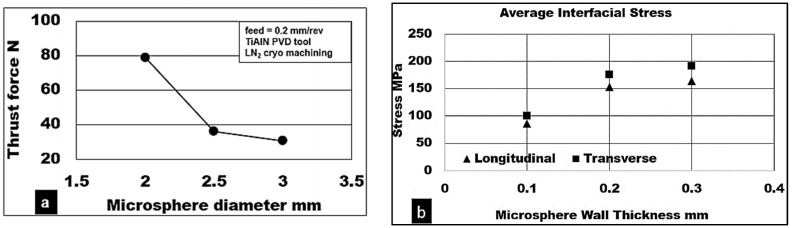

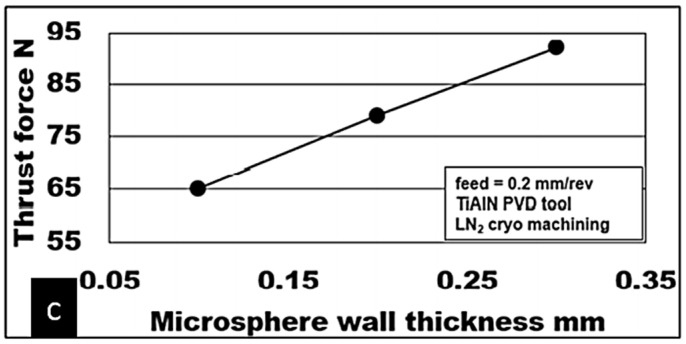
Microsphere damage model for drilling thrust force and von Mises stress.

In order to investigate the role played by the average microsphere diameter and shell thickness of the microsphere, drilling simulations were conducted at different values of microsphere geometries. Figure 6 represents the simulated thrust forces and von Mises stresses generated while cutting magnesium syntactic foam reinforced with different average microsphere diameters and shell thicknesses. From Figure 6a, it is observed that increasing the size of the microsphere diameter from 2 to 3 mm reduces the cutting forces by approximately 58%.

However, increasing the shell thickness of the microsphere increases the thrust force by about 30%. In addition, the simulation also shows the effect of shell thickness of the microsphere on the interfacial stresses developed in Figure 6c. It is noted that the longitudinal and transverse interfacial stresses along the magnesium matrix both increase with increasing shell thickness by approximately 50%. This result shows the significance of microsphere average size and shell thickness on the deformation characteristics of the syntactic foam.

**Figure 7:**
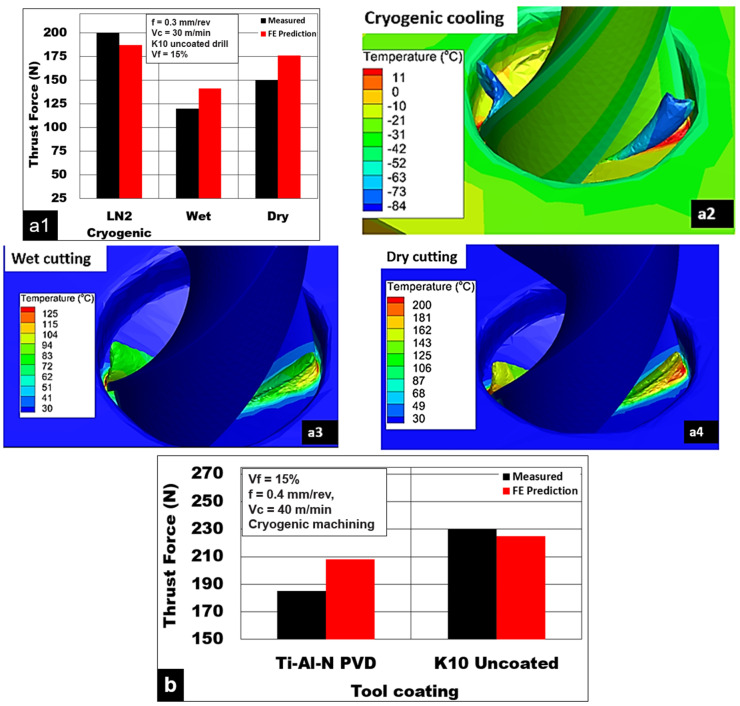
Influence of coolant type on (**a1**) thrust force. Simulated temperatures while cutting with (**a2**) cryogenic, (**a3**) Almag^®^ oil, and (**a4**) dry cutting. (**b**) Effect of drill coating on thrust force during machining of Mg-9Al-1.4Zn-15% hollow Al_2_O_3_ syntactic foam.

Cutting test results show the role of cooling/lubrication in the generation of thrust forces during the drilling of Mg-9Al-1.4Zn foams. Figure 7(a1) shows the measured thrust forces during cutting Mg-9Al-1.4Zn-15% hollow Al_2_0_3_ syntactic foams under three different cooling methods. As expected, cryogenic machining using liquid nitrogen generated the highest thrust forces (200 N), which were 33% higher than the dry machining (150 N). In general, of all cutting conditions, wet machining using the Almag^®^ mineral oil generated the lowest thrust force (120 N), which was 40% and 20% lower than cryogenic machining (200 N) and dry machining (150 N), respectively. The application of liquid nitrogen increases the hardness of the Mg-9Al-1.4Zn matrix. The brittle material behavior is promoted due to subzero temperatures in the cutting zone (Figure 7(a2)). Through mechanical testing under cryogenic conditions, it is shown that the yield compressive strength and peak compressive strength are increased while the ductility and strain to fracture of the magnesium foam are reduced [7,8]. This transition in deformation behavior to brittle type and a resultant increase in material hardness could be the possible reason for the higher magnitude of recorded thrust forces under cryogenic machining conditions [35]. Under cryogenic conditions, initiation and propagation of the reinforcement/matrix interface longitudinal crack and transverse matrix cracks favor the formation of discontinuous chips. A change in the deformation behavior of the material is shown through measured thrust forces with variations in cooling methods. The thrust force measured during dry machining was 25% higher than Almag^®^ oil. The presence of mineral oil in the cutting zone helps to minimize the adhesion of the magnesium matrix and reduce the generation of thrust force and temperature (Figure 7(a3)). The lubrication effect is maximized with the application of Almag^®^ oil, which helps to reduce BUE and friction. Thus, it is the preferred coolant for the light machining of magnesium alloys. Whereas during dry cutting, heat generated during shearing is dissipated into the cutting zone leading to the thermal softening of the magnesium matrix (Figure 7(a4)). This phenomenon promotes the adhesion of soft magnesium with increasing BUE formation and friction. Under these conditions, a highly plastic magnesium matrix is encouraged to yield much earlier than the alumina microsphere. This causes the load to be transferred through the interface leading to longitudinal cracks and reinforcement debonding [8].

Figure 7b depicts the influence of tool coating on measured force. As seen from the results, during cryogenic cooling conditions, drills with TiAlN PVD-coated tools generated 25% lower thrust forces than the uncoated K10 carbide drills. As discussed earlier, the use of liquid nitrogen tends to raise the hardness and compression strength of magnesium foam. Subzero cooling conditions enhance the brittle behavior of the magnesium foam. Cutting tool wear is shown to accelerate under cryogenic conditions [17]. The contribution to tool abrasion arises due to the rubbing of hollow Al_2_O_3_ in tandem with the hardened Mg-9Al-1.4Zn matrix. The presence of the tool coating reduces tool abrasion and helps to extend the tool’s life. The cutting edge is expected to wear rapidly due to high friction, the formation of BUE, and due to diffusion at higher temperatures. However, TiAlN PVD-coated tools possess a good nano hardness of 35 GPa, reduced coefficient of friction, and high-temperature operation (700 °C) [37]. This helps in the reduction in generated thrust forces while cutting with TiAlN PVD-coated cutting tools.

### 3.2. Surface Quality and Integrity

**Figure 8:**
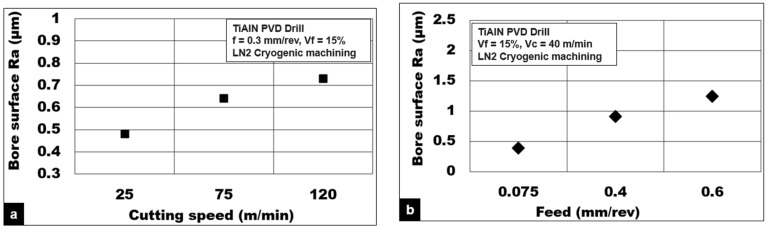
Influence of process parameters: (**a**) drilling speed and (**b**) feed on average surface roughness (Ra) during drilling Mg-9Al-1.4Zn-15% hollow Al_2_O_3_ foam.

Figure 8a,b show the effect of process parameters on achievable surface quality during cutting Mg-9Al-1.4Zn-15% hollow Al_2_O_3_ foam. An increase in average surface roughness Ra values by 52% is noticed with an increase in cutting speed from 25 to 100 m/min. Surface quality with an average roughness value of 0.48 µm was achieved at lower drilling speeds under cryogenic cooling conditions, which is considered a good surface finish. The average surface roughness (Ra) increased by 364% as the drill feed increases from 0.075 (0.39 µm) to 0.6 mm/rev (1.3 µm) during cryogenic cutting using a TiAlN-coated drill. The larger the feed, the higher the volume of metal removed, and the wider and deeper the feed marks, resulting in higher average surface roughness values.

**Figure 9:**
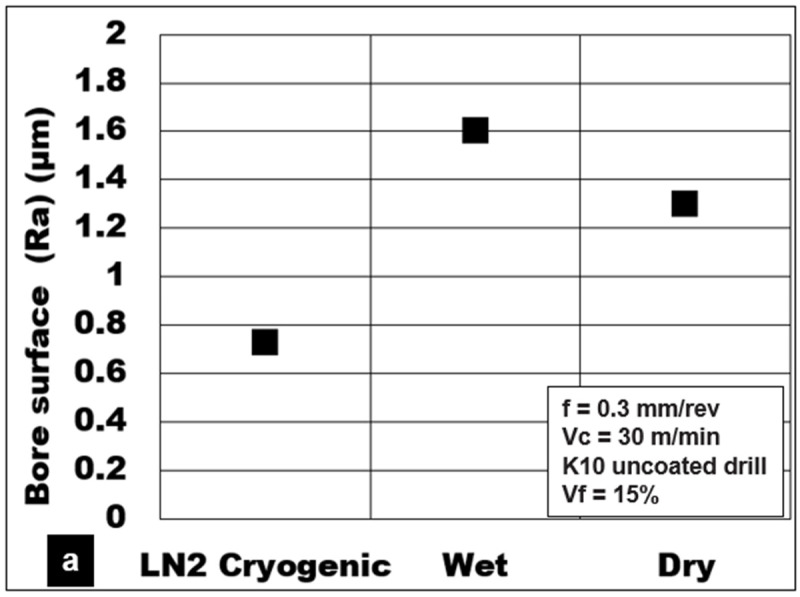

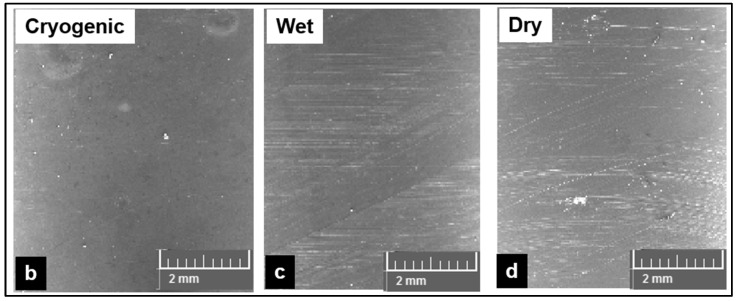
(**a**) Influence of coolant on average roughness during machining of Mg-9Al-1.4Zn-15% hollow Al_2_O_3_ foam. SEM images showing the bore surface under (**b**) cryogenic, (**c**) wet and (**d**) dry conditions.

The influence of different cooling methods on achievable surface quality during the drilling of Mg-9Al-1.4Zn-15% hollow Al_2_O_3_ foam using an uncoated K10 drill is shown in Figure 9a. The experiment results show that surface roughness values during cryogenic machining (0.73 µm) were reduced by approximately 55% and 43% compared to Almag^®^ oil (1.6 µm) and dry cutting (1.3 microns), respectively.

Thrust forces recorded during cryogenic machining were generally higher by 30% to 40% as compared to dry and Almag^®^ oil cutting. A drop in cutting temperature results in the hardening behavior of the magnesium foam. SEM investigation of the bore surface shows smaller feed marks, less machining-induced surface defects, and lower matrix plasticity in the form of material side flow (Figure 9b). Under these cooling conditions, the brittle nature of the material helps to produce smaller discontinuous chips resulting in good surface quality (Ra between 0.3 and 0.5 µm). During cutting using Almag^®^ oil, the effects of two-body tool abrasion and the sticking of Mg-9Al-1.4Zn to the cutting tool are reduced to a certain extent. However, the SEM investigation of the drilled bore surface shows certain areas comprising bore pits and voids. This is primarily attributed to the flushing away of the loose, brittle particles from the machined surface by the viscous mineral oil, which was applied under pressure (Figure 9c). Under dry cutting, the plasticity of Mg-9Al-1.4Zn is found to be the key deformation mechanism that affects the surface quality. The average surface roughness values measured on holes drilled under dry machining were 20% less than in wet machining conditions. Hole samples investigated using SEM showed slightly wider and deeper feed marks primarily due to material side flow. The rewelding of finer chips and smearing of the machined surface were some of the surface defects observed. An increase in shear zone temperature during dry cutting causes adhesion of finer pieces of chips resulting in deterioration of the surface quality (Figure 9d).

**Figure 10:**
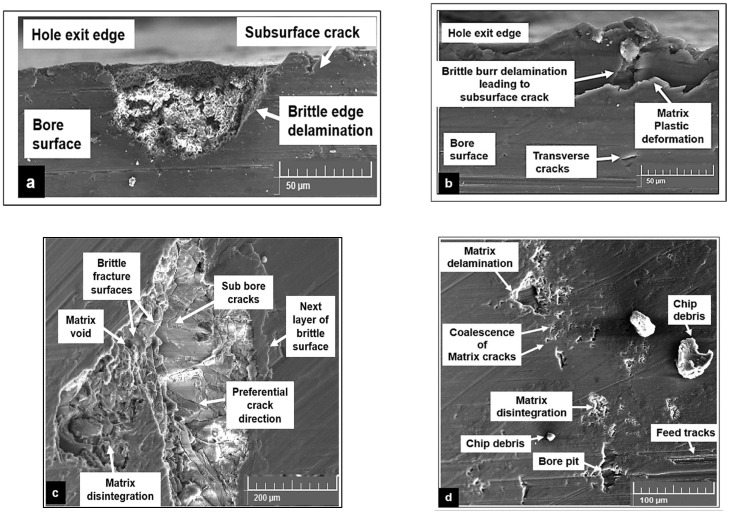
Some of the commonly found drilling-induced defects during machining of Mg-9Al-1.4Zn-based alumina syntactic foams: (**a**) Cryogenic. (**b**) Dry. (**c**,**d**) Wet Almag^®^ oil.

Figure 10 shows some of the commonly observed machining-induced defects during the drilling of Mg-9Al-1.4Zn magnesium reinforced with hollow alumina microspheres under different cooling conditions. This indicates the brittle nature of the material machined due to exposure to subzero cutting temperatures. On the other hand, wet machining using a viscous mineral oil produced surfaces that were comprised of flushed away particles leading to unexpected pits at certain locations on the bore section of the drilled hole (Figure 10c,d). This shows the significance of the application of mineral oil under pressure, which could affect the attainable surface quality and integrity by washing away the disintegrated particles on the drilled surfaces leading to empty spaces in the matrix. In addition to this, some locations on the hole exit side had delaminated edges and thin material push-off. During dry machining, the shear zone temperature allows plastic flow of the magnesium matrix around the ceramic microsphere, initiating interface debonding and preferential cracking (Figure 10b). Voids created by microsphere pullout from the matrix are closed at a faster rate due to accelerated densification of the magnesium matrix. [5]. The extent of plastic deformation undergone by the magnesium matrix is greater under dry machining compared to wet and cryogenic machining. This damage mechanism is visible in the form of smearing of side flow material along the tool feed tracks (Figure 10b).

**Figure 11:**
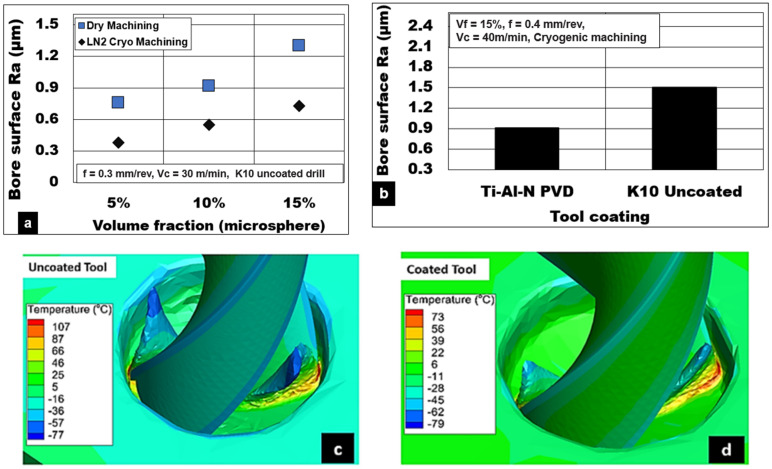
(**a**) Effect of hollow alumina volume fraction on bore surface roughness (Ra) during machining Mg-9Al-1.4Zn-based alumina syntactic foams; (**b**) effect of tool coating on Ra; (**c**,**d**) temperature contour while cutting with uncoated and coated tool using cryogenic cooling.

Figure 11a depicts the effect of volume fractions of hollow Al_2_0_3_ on the achievable surface quality during the drilling of Mg-9Al-1.4Zn foams. Under cryogenic machining conditions using an uncoated K10 drill, the average surface roughness value (Ra) increases by almost 92% as the volume fraction increases from Vf = 5% (0.38 µm) to Vf = 15% (0.73 µm). On the other hand, during dry machining with the same tool, the average surface roughness value (Ra) increases by 72% as the volume fraction increases from Vf = 5% (0.76 µm) to Vf = 15% (1.3 µm). The percentage of ceramic microspheres present in the magnesium matrix greatly affects the plastic deformation characteristics of the Mg-9Al-1.4Zn foam. In this work, within the range of foam volume fractions tested, an increase in the number of hollow Al_2_0_3_ in the matrix results in a foam that behaves like a brittle foam. The number of defect initiation points increases with volume fraction. Hence, the average surface roughness measured under cryogenic and dry drilling conditions correlates well with the SEM observation. Figure 11b shows the bore surface roughness values (Ra) during cryogenic machining of Mg-9Al-1.4Znbased 15% alumina foam. It is observed that the TiAlN PVD-coated tool (Ra: 0.91 µm) produces a surface with roughness values, which are 40% lower than the uncoated tool (Ra: 1.5 µm). This result shows the significance of tool coating in reducing friction due to the abrasion of ceramic microspheres and the adhesion of magnesium chips. The selected TiAlN PVD-coating is effectively shown to minimize the BUE and produce comparatively good surface quality and integrity. The temperature generation during using the coated tool under cryogenic cooling is at least 30 to 40 °C lower than the uncoated tool, as shown in Figure 11c,d.

### 3.3. Drilling Burr Formation 

**Figure 12:**
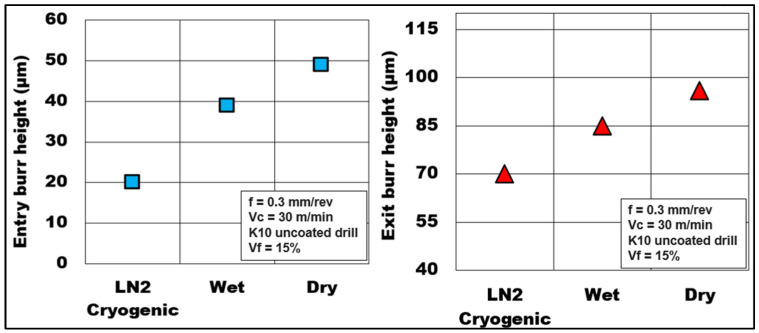
Effect of coolant on burr height during machining Mg-9Al-1.4Zn-based hollow alumina syntactic foams.

Burrs during plunge drilling are primarily formed due to thermo-mechanical loads that act on the material resulting in their push out as the tool exits the hole. Figure 12 presents the influence of the cooling method on the burr formation while cutting Mg-9Al-1.4Zn-based hollow alumina syntactic foams. Burr height was measured at the entry and exit side of the hole using a ZEISS^TM^ Smart proof confocal microscope, and the average value was recorded. It is observed that the burr height at the hole entry and hole exit side during cryogenic machining was 20 µm and 70 µm, respectively. These values are approximately 60% (entry) and 30% (exit) lower than the burr heights formed during dry machining. On the other hand, wet machining produced burrs, which were almost 95% (entry) and 22% (exit) higher than cryogenic machining and a 13–20% reduction compared to dry machining. In general, dry machining conditions produced higher values of burr height both at the entry (49 µm) and exit sides (96 µm) of the hole. During dry drilling, the heat generated in the cutting zone promotes plasticity of the matrix, thus causing material side flow. This is more pronounced during dry machining compared to wet and cryogenic machining conditions.

**Figure 13:**
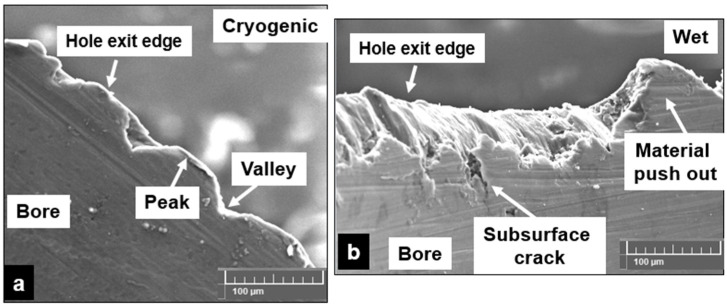

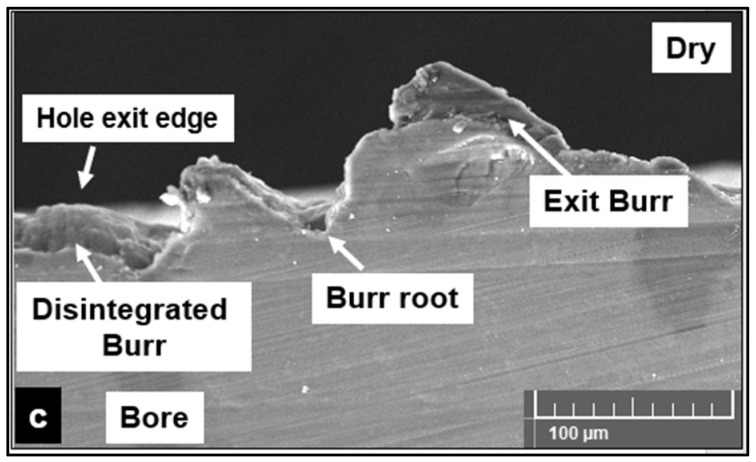
Different types of edges produced at the hole exit side of the Mg-9Al-1.4Zn-based hollow alumina syntactic foams: (**a**) Cryogenic. (**b**) Wet. (**c**) Dry. (Vc = 30 m/min, feed = 0.3 mm/rev, uncoated tool).

Investigations were carried out on the drilled burr forms produced using a VEGA 3 TESCAN SEM (Figure 13). It is noted that cryogenic machining using liquid nitrogen produces edges that are approximately sinusoidal in form. Due to a sudden drop in cutting temperature, the magnesium foam hardness is increased. This change in temperature conditions makes the magnesium matrix material behave more brittle than ductile. These conditions produce powdery chips due to material brittleness. This condition favors the formation of discontinuous chips. Due to increased brittleness, the areas around the hole edges tend to disintegrate. These machining-induced defects caused by cryogenic machining also develop as subsurface cracks as they propagate underneath the drilled surface. Small areas of the magnesium matrix that still show some signs of plastic deformation form small-scale burrs in some areas of the hole edge. However, for the majority of the magnesium matrix being exposed to subzero temperature conditions, brittle behavior is more dominant. This material behavior causes the burr root to disintegrate instantly and cause delamination of the edge. Mechanical testing carried out under cryogenic conditions on Mg-9Al-1.4Zn-based 15% hollow alumina syntactic foams showed a similar trend with reduced strain to fracture and decreased ductility at subzero test conditions [8].

This phenomenon is observed during cryogenic machining as reported in the literature [38]. Wet machining (Almag^®^ oil) provides effective lubrication to reduce tool abrasion and minimize the adhesion of magnesium chips. However, a major drawback in wet machining that is observed in this case is that the pressure coolant could cause the flushing away of the brittle surface layer. This surface layer is comprised of the deboned and fractured hollow spheres and brittle magnesium surface, as presented in Figure 13b. Although the majority of the measured values fall within an average roughness of 0.5 µm, the flushing effect causes satellite sites of large craters and microsphere pulled-out pits on the bore surface of the drilled hole. This aggravates the risk of subsurface damage to propagate beneath the drilled surface. In dry machining, the plasticity of the magnesium matrix is promoted due to the heat generated during the shearing process. This allows the matrix to flow around the hollow ceramic microspheres causing the material to yield under the applied shear load. This weakens the boundary interface between the metal and the ceramic reinforcement. The longitudinal cracks initiate and propagate to cause several locations of defect generation (Figure 13c). This allows the longitudinal cracks to coalesce with the transverse matrix cracks to constitute the shearing process. This causes the material to push out due to the combined effect of the thermal and shear load factors acting on the foam material during the drilling process. Thus, the height of burr formation during dry machining is comparably larger than in wet and cryogenic machining.

**Figure 14:**
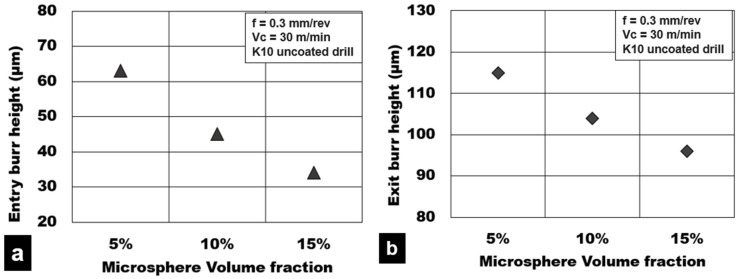
Effect of alumina volume fraction on burr height during machining Mg-9Al-1.4Zn-based hollow alumina syntactic foams under dry conditions: (**a**) Entry burr height and (**b**) exit burr height.

Figure 14a,b show the influence of hollow Al_2_O_3_ on the entry and exit burr height during dry drilling of Mg-9Al-1.4Zn syntactic foams. The test results show a decrease in burr height with increasing volume fraction. When the volume fraction increased from 5% to 15%, entry burr height and exit burr height decreased by 46% and 17%, respectively. Low volume fraction foams recorded an average entry and exit burr height of 63 µm and 115 µm, respectively. On the other hand, higher volume fraction foams with 15% vol of hollow alumina recorded average entry and exit burr heights of 34 µm and 96 µm, respectively. A decrease in burr height with increasing volume fraction points towards a reduction in the ductility of the syntactic foam. This is primarily attributed to an increase in the number of hollow microspheres in the matrix due to an increase in volume fraction for a given average size. The number of defect generation sites is increased. The strain hardening behavior of the magnesium matrix is enhanced due to the Hall–Petch effect of grain boundary pinning by the precipitates and the hollow microspheres. This causes a constraint on the matrix resulting in the higher hardness of the foam material. The yield strength of the material is enhanced. The magnesium matrix carries the applied load effectively with lower plastic deformation. Hence, the material push-out phenomenon is reduced in the case of 15% volume fraction foams. With an increasing number of ceramic microspheres, the number of defect generation sites also increases. The rapid joining of transverse matrix cracks with longitudinal interfacial cracks promotes crack propagation along a preferential direction and constrains the magnesium material side flow. There is a higher tendency for the brittle cracks to propagate and disintegrate the burr root. This causes delamination of the hole edge at a lower strain value compared to the more ductile low volume fraction foams. This phenomenon is attributed to cause a reduction in burr formation in higher volume fraction magnesium metallic foams.

### 3.4. Chip Morphology

**Figure 15:**
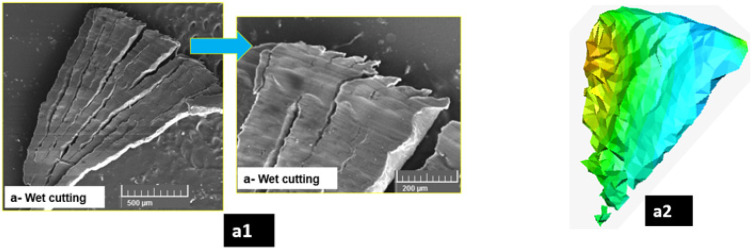

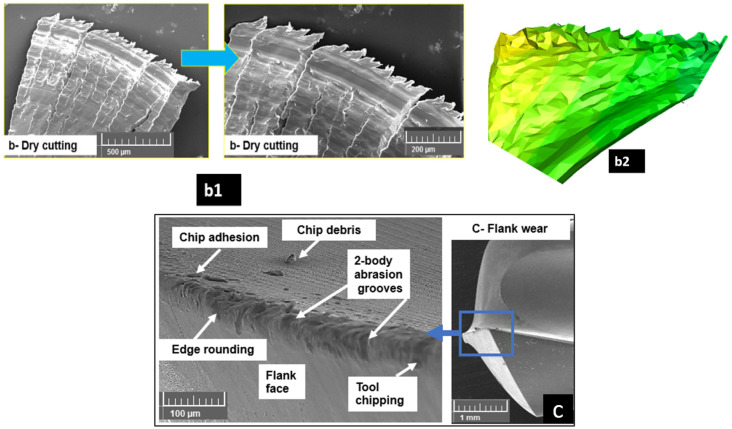
Chip morphology of Mg-9Al-1.4Zn hollow alumina foam: (**a1**,**a2**) experimental and FE predicted wet cutting chips; (**b1**,**b2**) experimental, and FE predicted dry cutting chips; (**c**) representative tool wear.

The influence of the lubrication method on the chip morphology during the drilling of Mg-9Al-1.4Zn syntactic foam is investigated through SEM micrographs (Figure 15). The significance of the cooling method on the achievable surface integrity during the drilling of magnesium syntactic foam has already been discussed in the previous section. The application of these novel magnesium syntactic foams in temporary biomedical stents and implants requires excellent surface quality and integrity. The study of chip formation is key to optimizing the drilling process design to achieve the required surface roughness. It has been shown that the surface roughness of the machined implant affects its corrosion behavior [19]. SEM investigation of chip forms shows that the type of chips formed during the drilling of magnesium foams were primarily discontinuous chips. Experiment chip morphologies during Almag^®^ oil and dry cutting of Mg-9Al-1.4Zn syntactic foams are shown in Figure 15(a1,b1).

As can be seen from the micrographs, the extent of brittle crack initiation and propagation is evident clearly on the smooth surface of chips. Chip serrations can be noticed clearly on the chip surface. The spacing between the serrations is approximately between 5–15 µm. After every 3 to 5 small serrations, a brittle matrix crack is evident that splits the chip, causing discontinuity. Past research has shown that the cutting tools used for cutting Mg-9Al-1.4Zn-based alumina syntactic foams are subjected to both two-body and three-body abrasion [7,8]. Wet machining using mineral oil is expected to provide lubrication and reduce the intensity of the abrasion of the tool surface by the ceramic microspheres and work-hardened matrix. The FE predicted chip morphology was very much in line with the experimental observation (Figure 15(a2)). The generation of high friction is expected to be lower while cutting with wet machining (Figure 7(a3)). Adhesion of the magnesium matrix is also expected to be reduced with the usage of mineral oil. Its heat-carrying ability induces localized brittle cracking of the matrix with the minimization of heat generation in the cutting zone. It has been discussed in the previous section that the application of mineral oil under pressure is detrimental to surface roughness. Loose, brittle layers become disintegrated, causing a higher percentage of machining-induced defects. The coalescence of surface defects is accelerated due to the application of pressure coolant. The cracks run almost along the full length of the chip along the transverse to the feed direction. The transverse crack initiation, coalescence, and movement are supported by the presence of voids, pores, and pits present in the matrix in large numbers. In addition, micro defects along the boundary region between the matrix and ceramic microspheres promote longitudinal crack initiation and propagation.

During dry machining, the plasticity of Mg plays an important role in the chip morphology. The rate of heat dissipation as a result of the shearing process into the workpiece material is higher during dry cutting (Figure 7(a4)). SEM micrographs of chips collected from the dry cutting show a serrated semi-continuous-type chip, as confirmed by FE predicted chip morphology (Figure 15(b2)). A small degree of edge serrations is noticed. The spacing between these small serrations is approximately the same size as the dry cutting. The transverse crack initiation and propagation are visible to a certain distance. Micrographs show regions of coalescence of transverse and longitudinal cracks. However, there are junctions where discontinuity in crack coalescence and propagation happens. It is observed that the termination of cracks due to the ductility of the magnesium material ahead of the crack leads to the closure of the crack. A higher degree of matrix ductility has caused the material side flow and push out. This results in higher friction and cutting force due to the formation of a built-up edge. A similar chip formation mechanism has been reported in the literature for metal matrix composites [9,39,40].

Characteristic tool wear during the drilling of Mg-9Al-1.4Zn syntactic foams is presented in Figure 15c during cryogenic machining. It is known that the hardness and brittleness of the magnesium foam are increased under cryogenic machining. Investigation of the tool surface shows areas of abrasive wear. The work-hardened matrix along with fractured hollow ceramic microspheres engage in both two-body and three-body abrasion. Abrasion wear tracks along with chipped edges indicate the brittle behavior of the magnesium syntactic foam while cutting under subzero cooling conditions. As a next step, tool life analysis will be conducted to characterize the acceleration of the tool wear and optimize the tool life during machining Mg-9Al-1.4Zn-magnesium syntactic foams.

## 4. Conclusions

Machinability studies were carried out on biodegradable Mg-9Al-1.4Zn-based hollow Al_2_O_3_ syntactic foams under different sustainable lubrication methods. A three-dimensional, thermomechanical finite element model was developed using AdvantEdge^TM^ software to simulate the drilling forces and cutting temperature generated. The attainable surface quality and integrity, drilling forces, chip morphology, and burr formation were investigated. Key observations made from this study:

1. Thrust force increased by almost 200% with an increase in the volume fraction of ceramic bubbles from 5% to 15% under cryogenic cooling conditions.
2. A reduction in cutting forces and temperature is observed using Ti-Al-N PVD-coated drills compared to uncoated drills under cryogenic machining by approximately 20% and 30%, respectively.
3. A 40% decrease in thrust force is observed with an increase in cutting speed from 25 to 120 m/min under cryogenic cooling. This is attributed to the effects of material thermal softening behavior.
4. An increase in the feed from 0.075 to 0.6 mm/rev results in higher thrust forces during cryogenic drilling (increase from 85 to 259 N). This is attributed primarily due to an increase in chip load and a higher percentage of ceramic bubble reinforcements in contact with the cutting tool.
5. Surface finish (Ra) showed a 45–55% improvement during cryogenic drilling of 15% syntactic foams with minimized subsurface damages compared to dry and wet cutting conditions. The higher the volume fraction, the higher the surface roughness (Ra).

## Figures and Tables

**Table 1:** Material Composition.

Average Al_2_O_3_ Size (mm)	Alumina	Iron Oxide	Calcium Oxide	Silica	Sodium Oxide	Density (g/cm^3^)
0.1–0.5 mm, 0.6–1 mm	99.7	0.006	0.013	0.026	0.27	1.700
Mg Alloy	87 Mg	9Al	1.4Zn	Cu ≤ 0.1%	Mn ≤ 0.13%	Si ≤ 0.5%

**Table 2:** Properties of Kennametal™ drill tools.

Geometry	Coated Drill	K10 Drill
**Grade**	Titanium–Aluminum Nitride fine grade	K10 CARBIDE
**Tool diameter (mm)**	5	5
**Number of flutes in tool**	3	3
**Shank diameter (mm)**	6	6
**Flute length (mm)**	20	35
**Point angle**	140°	130°
**Helix angle**	30°	30°

**Table 3:** Test factors.

Experiment Conditions		
Matrix	Mg-9Al-1.4Zn	
Reinforcement	Hollow Al_2_O_3_	
Microsphere (Volume fraction %)	5%, 10%, 15%	
Drilling speed (m/min)	40–120	
Feed (mm/rev)	0.075, 0.2, 0.4, 0.6	
Sample thickness (mm)	5	
Cutting insert	Kennametal™	TiAlN PVD coated K10-uncoated carbide
Lubrication	LN_2_ Cryogenic, Almag^®^ oil (Wet) Dry cutting	

## Data Availability

The data presented in this study are available on request from the corresponding author.
